# Can PIM-3 score predict risk for neonatal mortality vs. SNAPPE II and SNAP II scores in a tertiary surgical neonatal intensive care unit?

**DOI:** 10.3389/fped.2026.1933189

**Published:** 2026-10-14

**Authors:** Rachel Atkinson, Samiran Ray, Simon Hannam, Maria Chalia

**Affiliations:** 1 Neonatal Intensive Care Unit, Great Ormond Street Hospital for Children, London, United Kingdom; 2 Paediatric Intensive Care Unit, Great Ormond Street Hospital for Children NHS Foundation Trust, London, United Kingdom; 3 Department of Infection, Immunity and Inflammation, University College London, London, United Kingdom

**Keywords:** critical care, intensive care, mortality index, neonate, preterm

## Abstract

**Objective:**

To assess the accuracy of PIM-3 score in predicting neonatal mortality by comparing it to two established neonatal mortality scores, SNAPPE II and SNAP II.

**Study design:**

Retrospective study of all admissions to Great Ormond Street Neonatal Intensive Care Unit (NICU) for the calendar year of 2022. Individual scores were calculated for all admissions using the electronic health records and referring or transfer notes. Admissions <12 h and those with missing data were excluded. The sensitivity and specificity of each score at potential cut-off values were calculated and plotted as a receiver operator curve (ROC), by calculating the Area Under the Curve (AUC) and confidence intervals for each one. score. Subgroup analysis was used to assess the AUC of each score for gender, prematurity, age at admission, gestational weight, ventilation status, and whether the admission was planned.

**Results:**

The PIM-3 score had a higher AUC of 0.788 compared to SNAPPE II (0.738), and SNAP II (0.706), with optimal cut-off values at 9%, 30 and 11, respectively. The PIM-3 score performed better in preterm, lower birth weight and ventilated neonates. It also demonstrated a better correlation to length of stay compared to the other two scores, although statistically not significant.

**Conclusion:**

The PIM-3 score was equal to, or marginally better in predicting mortality in a mixed neonatal population compared to SNAPPE II and SNAP II. Further prospective analysis in a larger patient cohort is required to reproduce and validate its efficacy.

## Introduction

Scoring systems for predicting risk of mortality have been investigated for patients admitted on neonatal units. The commonest scores used in the neonatal population are CRIB II (Clinical Risk Index for Babies II) (1), SNAP II (Score for Neonatal Acute Physiology II) and SNAPPE II (SNAP Perinatal Extension II) (2) which include features that are more relevant to neonates, such as gestational age, birth weight, and Apgar score (3). Although the CRIB II score was simplified to include data from the first hour of admission as opposed to CRIB, which takes the first 12 h into consideration (4), it still only focuses on preterm neonates born at less than 32 weeks of gestation. The SNAP II score is also based on parameters in the first 12 h of admission to the neonatal intensive unit (NICU), such as mean blood pressure, lowest temperature, PaO2/FiO2 ratio, lowest pH, presence of multiple seizures, urine output, with the perinatal extension score (SNAPPE II) also including Apgar score, birth weight and being small for gestational age. The SNAPPE II score has recently been demonstrated to be the most accurate in predicting neonatal mortality (5). However, these scores have their limitations, as they are focusing on select clinical variables, not exploring primary diagnoses, or need for cardiopulmonary resuscitation or surgery prior to admission to NICU. Additionally, they are not routinely or widely employed in practice and are more likely to be used by individual units or as part of research projects. The paediatric mortality index III (PIM-3) score is utilised to predict mortality for patients admitted to a paediatric intensive care unit (PICU) and has evolved over the years to involve neonates being admitted to a PICU (6–8). Neonates may be admitted directly to a PICU because of a postnatal collapse at home or due to specialist services available, such as haemodialysis. There is also a neonatal population that is admitted to tertiary NICUs within paediatric hospitals, without a maternity unit on site. This neonatal subgroup is challenging to benchmark against other traditional NICUs or even against neonates admitted to PICUs. Nevertheless, the outcomes of these neonates may still be influenced by traditional neonatal factors such as gestation, and birth weight, as markers of physiological resilience, which are not adequately accounted for in the PIM-3 score.

It was, therefore, of interest to compare the PIM-3 score for this subgroup of neonatal patients against preexisting neonatal scoring systems. The PIM-3 score is based on clinical parameters in the first hour of admission, such as pupils, mechanical ventilation, systolic blood pressure, base excess (mmol/L), fractional inspired oxygen (FiO2), arterial partial pressure of oxygen (PaO2), as well as parameters relevant to the type of admission (planned vs. unplanned), high risk principal diagnoses, postoperative (cardiac vs. non cardiac), post cardiac bypass. Instead of allocating scores to a variable based upon the range in which it falls, it uses an algorithm which multiplies the value of each variable by a constant to produce a percentage. These constants have been calibrated based upon the correlation of that variable to mortality as found in development studies. Over time the values of the constants have been adjusted to account for changes in practice (see supplementary material). In recent studies, PIM-3 score was found to have an AUC for mortality of 0.77 (9) or 0.76 (10). A common counterargument, when using PIM-3 score for neonates, is that the gestational age and birth weight are not taken into consideration in assessing the mortality risk. Therefore, the aim this study was to compare the PIM-3 score against the neonatal mortality indices, SNAP II and SNAPPE II, and better understand if PIM-3 score is more accurate in predicting mortality.

## Methods

### Study design

Great Ormond Street Hospital is a specialised children's hospital without a delivery suite. All non-cardiac neonatal referrals of a corrected gestational age less than 44 weeks are managed on the NICU. There are infrequent admissions of older infants but less than 2 months of corrected gestational age with weight < 5 kg to facilitate patient flow between NICU and PICU and theatre capacity regarding surgical patients. This retrospective study reviewed all NICU admissions from 1st January 2022 until 31st December 2022.

The PIM-3 scores are routinely calculated upon admission to the NICU. The variables included in the score reflect the first hour of admission to the unit and the data is submitted to the national data collection network called PICANet on a weekly basis. The PIM-3 score performance was compared to the SNAP II and SNAPPE II scores, which reflect the first 12 h of admission to the neonatal unit.

### Data collection

Patients that were admitted during the period of interest were identified from the departmental admission books. Their demographics were cross referenced via the electronic patient record system (Epic, Epic Systems, WI, USA). Exclusion criteria included duplicate records created in error, patients with incomplete data precluding score calculation, and patients admitted to the NICU for less than 12 h. The latter was due to the fact that SNAP II and SNAPPE II scores are based on physiological data during the first 12 h of admission; therefore, patients with shorter than 12 h stay were excluded from the study (supplementary material). Patients who were discharged and re-admitted during this period, were included in the study and treated as independent admissions.

The relevant clinical data were obtained from the electronic patients' records. The demographic and clinical parameters collected were the following: admission and discharge date; admission cause; planned or unplanned admission; gestational age; Apgar scores; age and weight; whether they were small for gestational age; ventilation status; oxygen requirement; presence of seizures. In instances where the information was not available on the electronic health records, the transport notes and referral letters were also reviewed. The physiological variables used in the SNAPPE II and SNAP II indices (2) were also identified from the electronic patient records. The SNAPPE II and SNAP II scores were calculated using the Société Française d'Anesthésie et de Réanimation (SFAR) website (11). The existing PIM-3 scores, routinely calculated on admission for each patient were collected and used in the analysis (supplementary material).

### Data analysis

The analysis was conducted on SPSS, version 29.0.0.0 and presented as frequencies, percentages, and odds ratios; the Fisher's exact test was applied to identify any statistically significant differences in odds ratios of background variables between the groups which survived and died. For continuous variables, such as gestational age, weight, and length of stay, medians with interquartile ranges (IQRs) were calculated for both groups.

The sensitivity and specificity of predicting mortality at potential cut-off values for PIM-3, SNAP II, and SNAPPE II were estimated. These were plotted as a receiver operator curve (ROC), calculating the Area Under the Curve (AUC) and confidence intervals for each mortality score. The AUC was calculated as a measure to best assess how well each score performs in predicting mortality. Subgroup analysis was performed to assess the AUC for each mortality index within subgroups of gender, prematurity, age at admission, gestational weight, ventilation status, and whether the admission was planned. They were, subsequently, compared between groups using the MedCalc AUC comparison calculator (12, 13) for significance, which uses Delong's method (14).

The generated sensitivity and specificity tables were used and plotted against the relevant scores to visualise the increase of specificity and decrease of sensitivity along the scale, using the point where they intersect to identify potential cut-off values. This was supported by calculating the Youden Index using the formula: “sensitivity + specificity − 1”, which identified the highest possible combination of sensitivity and specificity and thus the optimal cut-off value.

The Spearman rank correlation coefficient between length of stay and mortality indices was calculated, in addition to the confidence intervals and *p* values for each result. The correlation coefficients were compared between the mortality indices to assess which performed better at predicting length of stay by using Fisher's r-z transformation (15). The level of statistical significance was determined as a *p* value less than 0.05. The confidence intervals were calculated at the 95% level.

## Results

### Patient characteristics

In the period of interest, 239 patients were identified from the NICU admission logbooks, of which 233 were indeed admitted to NICU (Figure 1). Following exclusion criteria, 36 patients were excluded from the study.; either because they had a NICU admission for less than 12 h (*n* = 20/233), did not have sufficient data (*n* = 2/233), did not have PIM-3 scores calculated on admission due to insufficient data entries at the time (*n* = 3/233), or did not sufficient birth history data to calculate the SNAPPE II score, which includes APGARs (*n* = 11/233).

**Figure 1:**
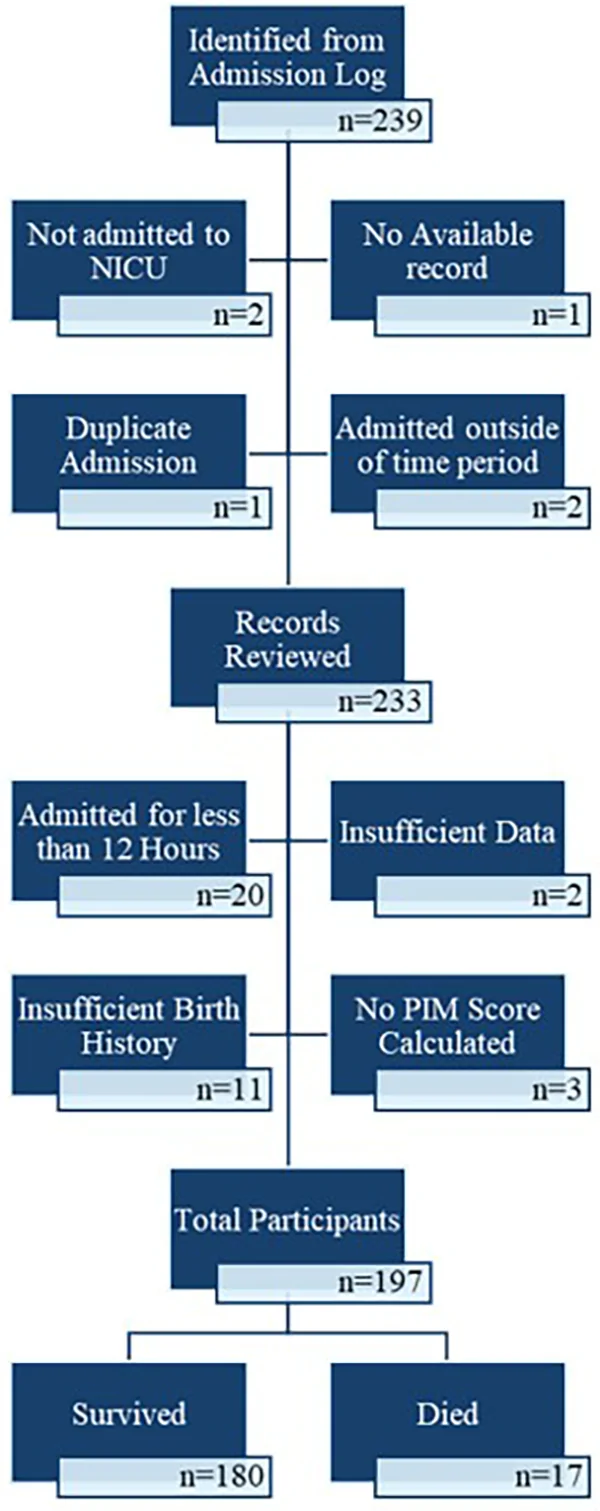
Patients’ recruitment.

In total, 197 patients were included in the study. Within this cohort, 180 neonates were alive at discharge, and 17 died. All the excluded neonates survived. The overall unit mortality rate was 7.3% (*n* = 17/233) and 8.6% for the population included in the study (*n* = 17/197). The median age on admission was 7 days of life (IQR: 1–33 days, *n* = 197). Amongst the 197 patients, there were 122 preterm neonates with a median gestational age at birth of 34 weeks and 3 days with a range of 22 weeks and 5 days to 36 weeks and 5 days. The median age at which preterm neonates were admitted to the unit was 15 days of life with a range of 1 day to 204 days of life (IQR: 2–43 days of life, *n* = 122). As seen on Table 1, 144 patients were less than 28 days old (*n* = 103/144 were admitted in the first week of life), and 53 patients over 28 days of life. All patients assessed were within the neonatal period, as in within 44 weeks of corrected gestational age, except for 4 patients ranging from 2 weeks to 8 weeks of life (corrected for gestational age) on admission. Of these 4 patients, there were 2 preterm neonates born at 22 weeks and 5 days and 24 weeks and 2 days respectively; one admitted for eye surgery and one for respiratory evaluation. There were no statistically significant differences in any of the background variables investigated between survivor and non-survivors (Table 1).

**Table 1:** Summary of patient characteristics and calculated odds ratios.

		Total *n* = 197		Deceased *n* = 17		Survived *n* = 180		Odds Ratio	*p* value
Gender	M	105	54%	9	53%	96	53%		
	F	90	46%	8	47%	82	47%	0.96	1
Term	Term	75	38%	3	18%	72	40%		
	Preterm	122	62%	14	82%	108	60%	0.32	0.11
Age	>28 days	53	27%	3	18%	50	28%		
	≤28 days	144	73%	14	82%	130	72%	0.56	0.57
Birth Weight	Normal	151	77%	15	88%	136	76%		
	<10th centile	46	23%	2	12%	44	24%	2.43	0.37
Elective	Planned	47	24%	2	12%	45	25%		
	Unplanned	150	76%	15	88%	135	75%	0.4	0.37
Ventilated	Unventilated	70	36%	10	59%	60	33%		
	Ventilated	127	64%	7	41%	120	67%	2.86	0.06

### Mortality index scores

The median PIM-3 score was 3.90% (IQR: 2.03%–8.77%) for those who survived and 16.13% (IQR: 8.72–29.48%) for those who died. For SNAPPE II the median score was 23 (IQR: 5–38.5) in the group that survived and 38 (IQR: 23–57) in those that did not. SNAP II had a median score of 5 (IQR: 5–16) in those that survived and 16 (IQR: 8.5–42.5) for those who died. The ROC for SNAPPE II was 0.738, for SNAP II it was 0.706 and PIM-3 it was 0.788 (Table 2 and Figure 2). The PIM-3 score provided the best balance of approximately equal sensitivity and specificity (Table 3), The SNAPPE II and SNAP II scores had lower specificities, 59% and 66% respectively, even at the optimal cut-off levels (Table 3).

**Table 2:** Area under the curve (AUC) and comparison to PIM-3 scores.

Index	AUC value	Confidence interval	*P* value	Comparison to PIM III	*P* value
SNAPPE II	0.738	0.632–0.845	0.010	−0.050	0.5675
SNAP II	0.706	0.546–0.848	0.005	−0.082	0.4077
PIM III	0.788	0.654–0.922	<0.001		

**Figure 2:**
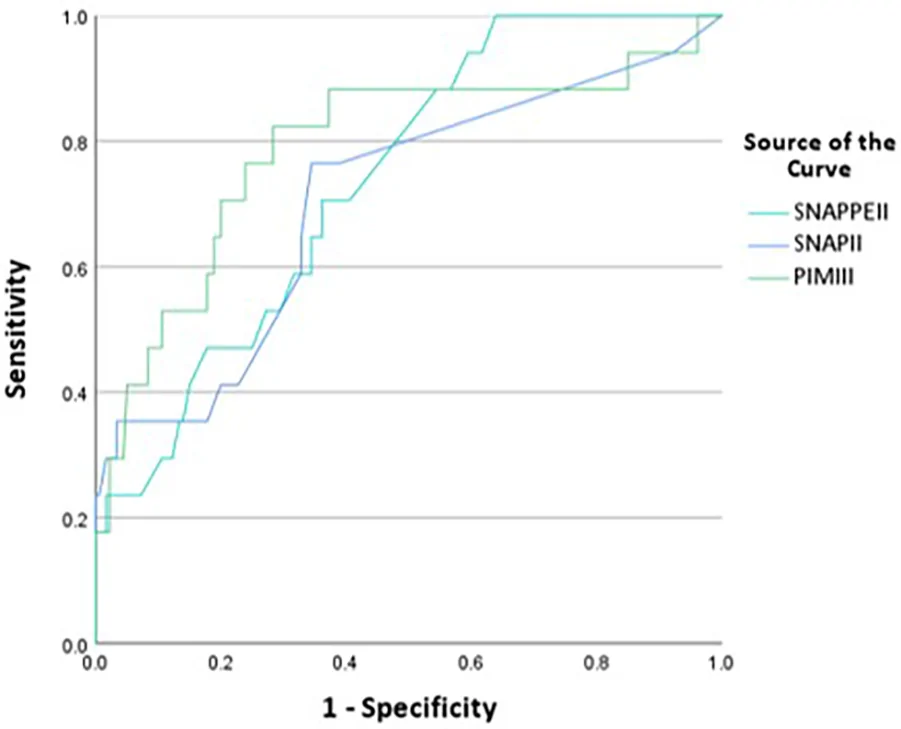
Receiver operatory curves for each mortality index.

**Table 3:** Cut-off values to maximise sensitivity and specificity for the mortality indices.

Index	Cut point	Sensitivity	Specificity	Youden
SNAPPE II	30	0.706	0.594	0.300
SNAP II	11	0.765	0.656	0.421
PIM III	9%	0.765	0.761	0.526

### Length of stay

The median length of stay (LOS) was 5 days (IQR: 2–15 days). For those who survived the median LOS was 4 days (IQR: 2–14 days) and for those who died the median LOS was 13 days (IQR: 1–22 days). The PIM-3 score had the highest a correlation coefficient of 0.394, vs. SNAP II and SNAPPE II (Table 4 & Figure 3) but none of the scores had a particularly high coefficient. SNAP II had a significantly lower correlation compared to PIM-3.

**Table 4:** Correlation coefficients for association between length of stay and the mortality indices.

Index	Correlation coefficient	Confidence interval	*P* value	Comparison to PIM III	*P* value
SNAPPE II	0.280	0.136–0.412	0.001	−0.087	0.3575
SNAP II	0.155	0.005–0.289	0.037	−0.212	0.0305
PIM III	0.367	0.230–0.490	0.001		

**Figure 3:**
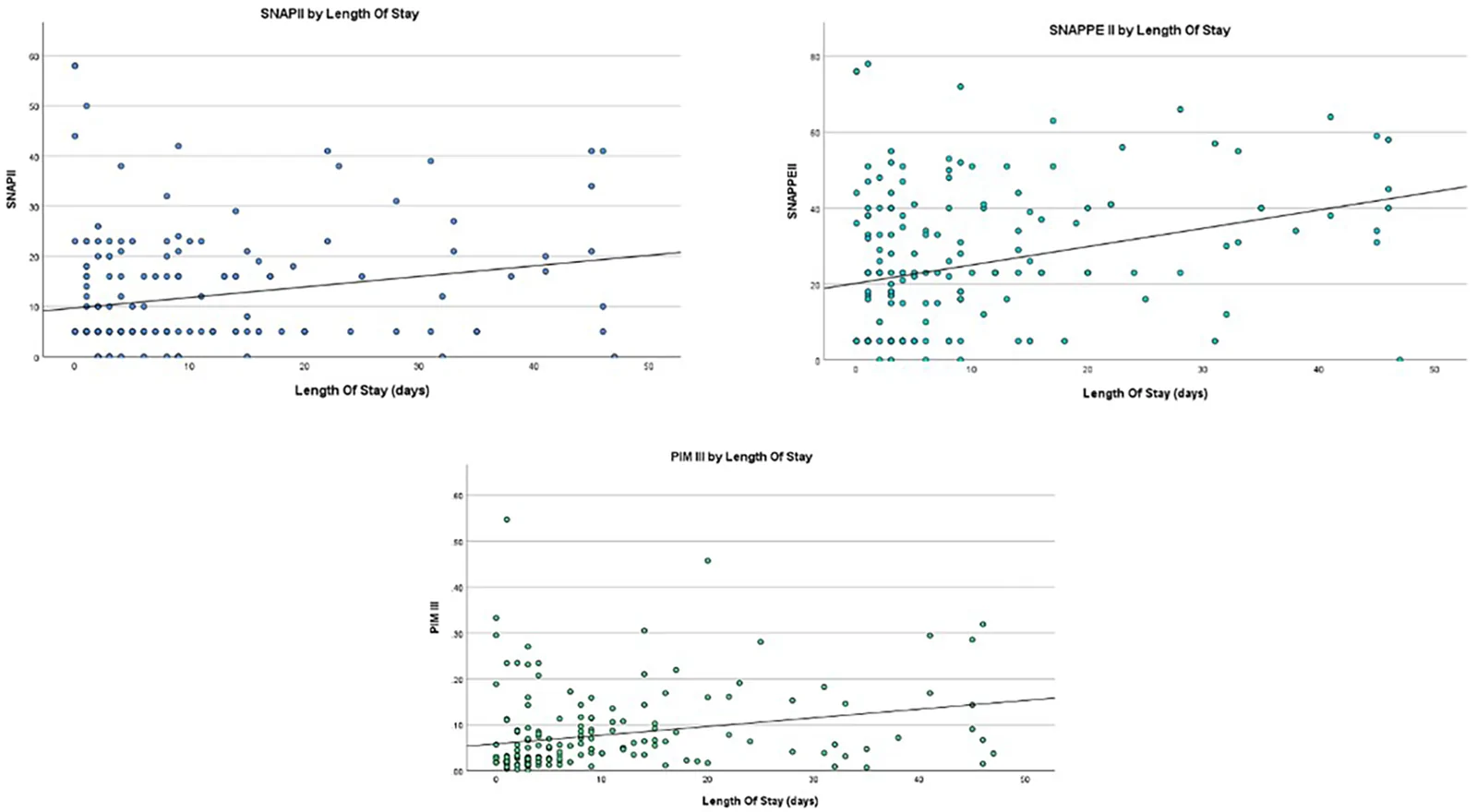
Scatter plots of length of stay in relation to mortality index scores.

### Sub-group analyses

Amongst the 197 patients, there were 122 preterm and 75 term neonates. In preterm neonates, the PIM-3 score offered the largest AUC of 0.794 (*p* *<* 0*.*0001); SNAP II had a statistically significant lower AUC (0.657), compared to PIM-3 (*p* *=* 0*.*022). There were no significant differences between SNAPPE II and PIM-3. In term neonates, the SNAPPE II score offered the highest AUC amongst term neonates at 0.907 (*p* *=* 0*.*02), with SNAP II, AUC 0.852 (*p* *=* 0*.*04) coming second and outperforming PIM-3, AUC 0.634 (*p* *=* 0*.*43).

Amongst the small for gestational age neonates, only the PIM-3 AUC, at 0.932 (*p* *=* 0*.*04), was statistically significant; SNAPPE II AUC at 0.790 (*p* *=* 0*.*17); SNAP IIs at 0.672 (*p* = 0*.*404). In non-SGA neonates, all three scores offered statistically significant indications of mortality risk, with AUCs of 0.722 (*p* *=* 0*.*005) for SNAPPE II, 0.708 (*p* *=* 0*.*008) for SNAP II and 0.769 (*p* *=* 0*.*001) for PIM-3.

In unplanned neonatal admissions, there were no statistically significant differences between the AUCs for each score, with the SNAPPE II AUC of 0.736 (*p* *<* 0*.*001), SNAP II at 0.707 (*p* = 0*.*009) and PIM-3 at 0.849 (*p* *<* 0*.*001). In planned admissions, none of the scores had significant AUCs, PIM-3 at 0.411 (*p* *=* 0*.*673), SNAPPE II at 0.611 (*p* *=* 0*.*598), and SNAP II at 0.706 (*p* *=* 0*.*330) or any significant differences between them.

Amongst the non-ventilated neonates, SNAPPE II, SNAP II, and PIM-3 scores performed similarly, with no statistically significant differences between them, with SNAPPE II having an AUC at 0.752 (*p* *=* 0*.*11), SNAP II, an AUC at 0.788 (*p* *=* 0*.*004) and PIM-3, an AUC at 0.752 (*p* *=* 0*.*11) respectively. Within the ventilated neonates, only the PIM-3 score offered a significant AUC value of 0.858 (*p* *=* 0*.*002). This was significantly higher than the SNAP II AUC of 0.601 (*p* *=* 0*.*37) was, and statistically significant in comparison to PIM-3 (*p* *=* 0*.*04). The SNAPPE II AUC was 0.693 (*p* = 0.09), but not statistically significant in comparison to PIM-3 (*p* *=* 0*.*07).

## Discussion

In this study, the predictive abilities of three differing mortality scores were compared for admissions onto a free standing neonatal intensive care unit within a specialised children's hospital. In this cohort, the mortality rate was 8.6%, similar to previously recorded UK values (16). The PIM-3 score provided the best balance between sensitivity and specificity when compared with SNAPPE II and SNAP II.

All three indices achieved AUC values above 0.7, suggesting acceptable discriminatory ability for predicting mortality. None of the indices, however, exceeded an AUC of 0.8, indicating that although useful, their respective predictive performances remained imperfect in this complex surgical neonatal population. The PIM-3 score demonstrated the highest AUC at 0.788, followed by SNAPPE II at 0.738 and SNAP II at 0.706. Despite this, the confidence intervals overlapped substantially, reflecting the relatively small sample size and low number of deaths within the cohort. Consequently, no statistically significant superiority of one score over another could be demonstrated. Nevertheless, PIM-3 consistently performed at least equally well, and in several analyses marginally better, than the neonatal-specific scores.

The comparatively good performance of PIM-3 was notable given that it does not include gestational age, birth weight, or Apgar scores in its calculation. These variables are traditionally considered significant indicators of neonatal mortality and are incorporated within SNAPPE II. One possible explanation is that mortality within a tertiary surgical neonatal population may be more heavily influenced by acute physiological instability and severity of underlying pathology than by perinatal characteristics alone. Surgical conditions such as necrotising enterocolitis, severe intraventricular haemorrhage, congenital diaphragmatic hernia, complex airway abnormalities, gastrointestinal malformations, and severe intracranial pathology may contribute substantially to mortality risk irrespective of gestation or birth weight. For instance, necrotising enterocolitis and intracranial haemorrhage are two high-risk diagnoses, that if present, are considered in the calculation of PIM-3 score and would significantly inform the mortality risk, as opposed to the SNAP II and SNAPPE II that do not include primary diagnoses in their calculation. Similar observations have been reported in PICU populations where acute physiological instability was more strongly associated with mortality than demographic variables alone (17).

To our knowledge, SNAPPE II and SNAP II have not previously been directly compared with PIM-3 in a neonatal surgical intensive care setting. This comparison is clinically relevant because specialised children's hospitals increasingly manage neonates separately from general neonatal networks, often in units integrated within paediatric tertiary referral systems. In such environments, traditional neonatal scoring systems may not fully reflect the complexity of surgical and post-operative neonatal disease. Furthermore, some of these neonates are admitted beyond the first or second week of life and not immediately after birth. When admission occurs beyond the first week of life, the neonate has undergone substantial physiological transition from the immediate postnatal period, potentially altering the clinical presentation and the underlying physiological parameters. Traditional neonatal scores have been designed to reflect this immediate transition to postnatal life rather than underlying disease complexity, and, therefore, underperformed in this cohort. The finding that PIM-3 performed similarly, and in few subgroups better, than neonatal-specific scores suggests that it may have applicability in surgical neonatal populations, such as in this cohort.

The performance of SNAPPE II and SNAP II in this study was lower than that reported in their original validation cohorts. Richardson et al. reported an AUC of approximately 0.91 for SNAPPE II (2), while subsequent studies have described AUC values ranging between 0.80 and 0.99 depending on patient population and case mix (18–20). Similarly, previous studies evaluating SNAPPE II in very preterm neonatess demonstrated strong predictive performance for mortality and severe morbidity (21, 22). The lower discriminatory ability observed in this cohort may reflect important differences in patient demographics and referral patterns. Unlike other neonatal validation studies, this cohort included transferred, post-operative neonates, or older neonates admitted from home or other centres beyond the immediate postnatal period. Such patients may exhibit physiological disturbances and mortality risks that are not adequately captured by scoring systems developed predominantly for early neonatal intensive care admissions.

The performance of PIM-3 in this cohort was broadly consistent with previously published paediatric intensive care studies. Meta-analyses and external validation studies have reported pooled AUC values between 0.75 and 0.88 (10, 23, 24). As no studies have previously assessed the predictive ability of PIM-3 in neonates, the fact that this score had the strongest predictive ability for mortality when compared to neonatal specific scores, may suggest that PIM-3 can be explored and utilised across neonatal units with a similar complex, surgical case mix to the one of this present study. Although PIM-3 is not neonatal-specific, its reliance on early physiological instability, respiratory support, and high-risk diagnostic categories may make it more adaptable to a heterogeneous tertiary surgical neonatal population. Furthermore, because PIM-3 is calculated within the first hour of admission, it may capture the degree of critical illness prior to intensive care interventions and may be more advantageous in rapidly evolving neonatal surgical conditions where early risk stratification is particularly valuable.

The optimal cut-off value for SNAPPE II in this study was 30, which provided a sensitivity of 71% and a specificity of 59%, (Youden index 0.3). This was also shown by Mia et al. (25), although other studies have identified lower optimal thresholds, particularly in more general neonatal populations (19, 20). The optimal cut-off value for SNAP II was 11, like that found in the literature (19) and was associated with similar sensitivities, but a lower specificity and thus a lower Youden score. The optimal cut-off value of PIM-3 was 9%, with a sensitivity of 77% and a specificity of 76%, Youden index 0.53. The PIM-3 score offered the highest combined sensitivity and specificity at its optimal cut-off point for predicting mortality, however, SNAP II outperformed SNAPPE II. Although the findings in this cohort suggest that PIM-3 may provide superior balance between false-positive and false-negative classifications, the reported cut-off values were calculated from the same dataset, and this significantly limits their performance in any other population. These cut-off values can only be used as a preliminary guidance and cannot, therefore, be universally applied without further external validation in a much larger sample size.

All three scores showed statistically significant but relatively weak correlations with LOS. PIM-3 demonstrated the strongest correlation coefficient, although the relationship remained overall modest. This likely reflected the multifactorial determinants of neonatal intensive care LOS which includes surgical recovery, establishment of feeding, chronic respiratory morbidity, discharge planning, and social factors, which are not fully captured by physiological severity scores alone. Nevertheless, the stronger correlation observed with PIM-3 may indicate that early physiological instability is associated with more prolonged admissions in tertiary neonatal surgical care. Previous studies have similarly identified SNAPPE II as a predictor of prolonged hospitalisation (26), while SNAP II has shown weaker associations with length of stay (27). This suggests that PIM-3 would be the most relevant score to use when considering admission planning.

Subgroup analyses identified important differences in score performance according to patient characteristics. The PIM-3 score performed better in preterm neonates and was the only score to demonstrate statistically significant mortality discrimination in this subgroup, despite other studies finding SNAPPE II to be a good indicator of mortality in preterm neonates with an AUC of 0.78 (22). This finding was somewhat unexpected given the absence of gestational age within the PIM-3 algorithm. However, it is possible that preterm neonates requiring tertiary surgical or specialist intensive care represent a particularly physiologically unstable population, in whom acute illness severity outweighs baseline prematurity as a determinant of mortality. In addition, these preterm had a median age on admission of 15 days (IQR: 2–43 days), which highlights the fact that their physiology is different to the immediate postnatal one, and factors, such as ventilation days, co-existing brain injury, haemodynamically significant patent ductus arteriosus may alter their response to the underlying diagnosis leading them to the specialised surgical NICU. Conversely, SNAPPE II and SNAP II performed particularly well in term neonates, likely reflecting the greater relevance of perinatal variables such as Apgar scores and birth characteristics within this group. The strong performance of PIM-3 in ventilated and small-for-gestational-age neonates was also notable. In ventilated neonates, PIM-3 was the only score to achieve statistically significant mortality discrimination. This may relate to the incorporation of mechanical ventilation and oxygenation variables directly into the score calculation, allowing better identification of severe respiratory failure and cardiorespiratory instability. Similarly, the strong PIM-3 AUC of 0.932 observed in small-for-gestational-age suggests that acute physiological compromise has greater prognostic value than anthropometric parameters alone in this cohort, as opposed to previous studies which have shown SNAPPE II as a good predictor of mortality (with an AUC of 0.84) in this population (21). These findings should be considered carefully, however, as the sample size was small with only 46 neonates being small for gestational age in this cohort.

As expected, all three scores performed poorly in planned admissions. These patients are generally physiologically stable at the time of admission despite requiring complex procedures or specialist care. Since all three indices are primarily designed to assess acute physiological derangement, their reduced utility in elective admissions was unsurprising and highlighted the limitations of applying illness severity scores to lower acuity populations.

There were several limitations in this study. This was a retrospective single-centre study with a relatively small sample size and low number of mortality events which limited statistical power and widened confidence intervals. Additionally, the calculation of SNAP II and SNAPPE II depended heavily on the completeness and accuracy of retrospective documentation. Variables such as urine output and Apgar scores were inconsistently recorded, introducing further potential measurement bias. Furthermore, the inclusion of repeated admissions may have skewed the true sensitivity and specificity of the scores, as these patients must have survived at least one admission potentially impacting their likelihood of surviving another, however, this choice was made to reflect real-time clinical practice, where neonates frequently require multiple admissions for separate clinical events.

Another limitation was that the case mix reflected a highly specialised tertiary surgical neonatal population, not always admitted immediately after birth, transferred in from other centres and often admitted further away from the immediate postnatal transitional period. Therefore, this study may not be generalisable to conventional NICUs attached to a maternity unit with a delivery suite. Nevertheless, this unique patient population represents an increasingly important subgroup within specialised paediatric centres, and currently available mortality prediction tools have not been extensively evaluated in this setting.

Overall, these findings suggest that PIM-3 is at least comparable, and potentially superior, to SNAP II and SNAPPE II for mortality prediction within a tertiary surgical NICU in a children's specialised hospital. Its ease of calculation, early applicability, and consistent performance across higher-risk subgroups supports its use for benchmarking and clinical audit purposes in specialised NICU settings, especially as more surgical NICUs, separate from maternity services, are being created within specialised children's hospitals in and outside of the UK. Larger multicentre prospective studies are required to validate these findings and to determine whether adaptation of paediatric scoring systems for neonatal surgical populations may further improve prognostic accuracy.

## Data Availability

The original contributions presented in the study are included in the article/Supplementary Material, further inquiries can be directed to the corresponding author/s.
