# Supplementary material for: Can PIM-3 score predict risk for neonatal mortality vs. SNAPPE II and SNAP II scores in a tertiary surgical neonatal intensive care unit?
Source: Front Pediatr. 2026 Oct 14;14:1933189. doi: 10.3389/fped.2026.1933189 (PMC13713864; doi:10.3389/fped.2026.1933189)
Supplement: Supplementary file 1 [file Supplementaryfile1.docx]

**Supplementary Material**

**SNAP II Score & SNAPPE II Score**

The table below describes the calculation of the Score for Neonatal Acute Physiology II. The addition of the Apgar score, the birth weight and the small for gestational age parameters constitute the perinatal extension of the score (SNAPPE II).

**PIM-3 score**

The paediatric index of mortality 3 is calculated using a regression formula (see table below) based on physiological and clinical variables reflecting the first hour of admission to intensive care:

Physiological variables:

Pupillary reaction, mechanical ventilation or non-invasive ventilation, systolic blood pressure, fraction of inspired oxygen requirement, saturations, partial pressure of oxygen (only from an arterial sample), base excess (as measured from the first blood gas obtained in the first hour of admission).

Clinical variables:

Elective admission: Yes – No.

Main reason for admission:

- Not recovery from surgery or procedure
- Recovery post-bypass procedure
- Recovery post non-bypass cardiac procedure
- Recovery post non-cardiac surgical procedure

Very high-risk primary diagnosis:

- None
- Cardiac arrest preceding admission to intensive care
- Severe combined immune deficiency
- Leukaemia or lymphoma after first induction
- Liver failure

High risk primary diagnosis:

- None
- Spontaneous cerebral haemorrhage
- Cardiomyopathy or myocarditis
- Hypoplastic left heart syndrome
- Neurodegenerative disorder
- Necrotising enterocolitis

Low risk primary diagnosis:

- None
- Asthma
- Croup
- Bronchiolitis
- Obstructive Sleep Apnoea
- Diabetic ketoacidosis
- Seizure disorder
